# Age-adjusted Charlson comorbidity index in recurrent glioblastoma: a new prognostic factor?

**DOI:** 10.1186/s12883-021-02532-x

**Published:** 2022-01-21

**Authors:** Melanie Barz, Stefanie Bette, Insa Janssen, A. Kaywan Aftahy, Thomas Huber, Friederike Liesche-Starnecker, Yu-Mi Ryang, Benedikt Wiestler, Stephanie E. Combs, Bernhard Meyer, Jens Gempt

**Affiliations:** 1 Department of Neurosurgery, Technical University Munich, School of Medicine, Klinikum rechts der Isar, Munich, Germany; 2 Department of Neuroradiology, Technical University Munich, School of Medicine, Klinikum rechts der Isar, Munich, Germany; 3 Department of Radiology and Neuroradiology, University of Augsburg, Augsburg, Germany; 4 Department of Neurosurgery, Hôpitaux Universitaires de Genève, Geneva, Switzerland; 5 Institute of Clinical Radiology and Nuclear Medicine, University Medical Center Mannheim, Medical Faculty Mannheim, Heidelberg University, Mannheim, Germany; 6 Department of Neuropathology, Technical University Munich, School of Medicine, Klinikum rechts der Isar, Institute of Pathology, Munich, Germany; 7 Department of Neurosurgery, Helios Klinikum Berlin Buch, Berlin, Germany; 8 Department of Radiation Oncology, Technical University Munich, School of Medicine, Klinikum rechts der Isar, Munich, Germany; 9 Department of Radiation Sciences (DRS) Helmholtz Zentrum Munich, Institute of Innovative Radiotherapy (iRT), Munich, Germany; 10 German Cancer Consortium (DKTK), Partner Site Munich, Munich, Germany

**Keywords:** Age-adjusted Charlson comorbidity index, Recurrent glioblastoma, Surgery, Prognostic factor

## Abstract

**Background:**

For recurrent glioblastoma (GB) patients, several therapy options have been established over the last years such as more aggressive surgery, re-irradiation or chemotherapy. Age and the Karnofsky Performance Status Scale (KPSS) are used to make decisions for these patients as these are established as prognostic factors in the initial diagnosis of GB. This study’s aim was to evaluate preoperative patient comorbidities by using the age-adjusted Charlson Comorbidity Index (ACCI) as a prognostic factor for recurrent GB patients.

**Methods:**

In this retrospective analysis we could include 123 patients with surgery for primary recurrence of GB from January 2007 until December 2016 (43 females, 80 males, mean age 57 years (range 21–80 years)). Preoperative age, sex, ACCI, KPSS and adjuvant treatment regimes were recorded for each patient. Extent of resection (EOR) was recorded as a complete/incomplete resection of the contrast-enhancing tumor part.

**Results:**

Median overall survival (OS) was 9.0 months (95% CI 7.1–10.9 months) after first re-resection. Preoperative KPSS > 80% (*P* < 0.001) and EOR (*P* = 0.013) were associated with significantly improved survival in univariate analysis. Including these factors in multivariate analysis, preoperative KPSS < 80 (HR 2.002 [95% CI: 1.246–3.216], *P* = 0.004) and EOR are the only significant prognostic factor (HR 1.611 [95% CI: 1.036–2.505], *P* = 0.034). ACCI was not shown as a prognostic factor in univariate and multivariate analyses.

**Conclusion:**

For patients with surgery for recurrent glioblastoma, the ACCI does not add further information about patient’s prognosis besides the well-established KPSS and extent of resection.

## Background

Glioblastoma (GB) multiforme is the most aggressive primary brain tumor in adults with a median age of 64 years at the time of diagnosis [1, 2]. Although there have been therapeutic advances over the last decades, the local progression within weeks to months is the main reason for treatment failure [3–5]. In the last years, more therapy options have been developed for recurrent disease, such as more aggressive surgery, re-irradiation, or chemotherapy [6–8]. The Karnofsky Performance Status Scale (KPSS) is one of the most important prognostic factors regarding the overall survival (OS) of newly diagnosed GB patients. In addition to the patients’ age [9, 10], it is used for decision-making in recurrent GB as well. Younger patients who have obtained functional independence benefit more from recurrent surgery than older patients with a reduced KPSS. Besides age and KPSS, complete resection stands out as the most important prognostic factor [11, 12]. Due to demographic changes within the expanding elderly population, an increasing incidence rate of GB and recurrent GB among elderly people has been observed [1]. The patients’ increasing age is usually associated with a higher rate of comorbidities and this burden can be assessed by the Charlson comorbidity index (CCI), a validated score to estimate mortality in patients with multiple comorbidities [13–15]. In 1994, this could be extended by adding the age, so that the age-adjusted CCI was created [16]. Although the (age-adjusted) CCI initially proved its worth in internal diseases, in recent studies it has also proved to be a useful tool for tumour diseases in addition to the well-known and established KPSS. Initial steps in this direction have been taken by various workgroups in the case of relapsing tumors of different entities. So, the group of Martinez could show that in the case of relapsed Hodgkin’s lymphoma in patients older than 50 years, a CCI > 1 is associated with poor OS and progression-free survival, independently of age [17]. Other publications that cover relapsing neck cancer have produced similar results: If the frequency or severity of comorbidity increases, survival progressively decreases [18, 19]. The aim of this study was to evaluate patient comorbidities by using the age-adjusted Charlson Comorbidity Index (ACCI) as a prognostic factor in recurrent GB.

## Methods

This retrospective, non-interventional bicentric study was approved by the medical ethics committee of the Technical University Munich (5625–12) and is in accordance with the ethical standards of the 1964 Declaration of Helsinki and its later amendments [20].

### Patient population

**Fig. 1:**
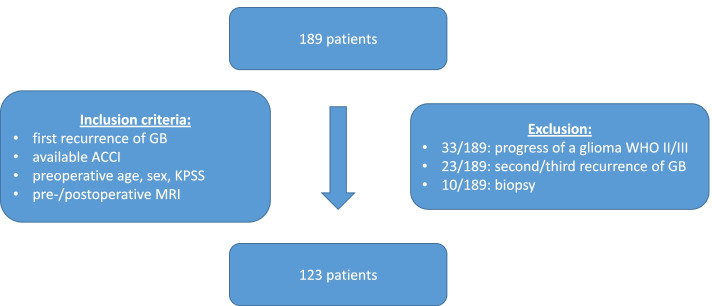
Flowchart of patient-selection process

**Fig. 2:**
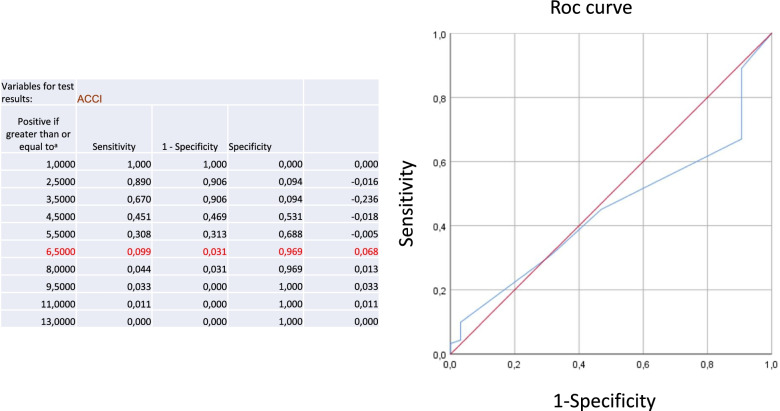
Roc curve analysis and use of Youden index: ACCI > = 6.5 there is a specificity of 96.9% and a sensitivity of 9.9

From 189 possible eligible patients, 33 were excluded due to progression from a lower grade of glioma and another 23 patients were excluded because of second or third recurrence, 10 were excluded due to biopsy. 123 consecutive patients with recurrent GB (WHO IV) between January 2007 and December 2016 met our inclusion criteria: surgery for primary recurrence of GB with available ACCI, preoperative age, sex, KPSS, and pre−/postoperative MRI. We retrospectively reviewed all the clinical data of these 123 patients and analyzed the comorbidities by using the age-adjusted ACCI (Fig. 1). In detail, the CCI takes into account 19 conditions with a score for each comorbidity from 1 to 6, depending on the risk of death. By using the age-adjusted form of the CCI, the score additionally receives the specific weighting of age. This means that a 50-year-old patient receives one additional point for every decade (e.g., in 50–59 years, 1 point; 60–69 years, 2 points; 70–79 years, 3 points), and these age points are added to the CCI score (e.g., 0, 1, 2, 3, etc.) [14, 16]. Using a Roc curve analysis and the Youden index, we were able to detect a cut-off for the ACCI at 6.5 (Figure). Additionally, we analyzed preoperative sex and functional status quantified by the KPSS. Recurrence and progression were evaluated by MRI using the RANO criteria [21]. Recurrent neurosurgical resection was performed using intraoperative neuromonitoring, neuronavigation, and 5-aminolevulinic acid (ALA) with the aim of maximum resection of the contrast-enhancing tumor part. In patients with inoperable tumor recurrence, a biopsy was performed for histopathological diagnosis. Patients with biopsy were not included in this study. Within the first surgery, molecular pathological findings were analyzed according to the WHO criteria of 2016 [22] (i.e., O-6-methylguanine-DNA-methyltransferase [MGMT] promotor methylation (35/123; of which MGMT methylation could be detected in 15 patients [23], mutation status of isocitrate dehydrogenase [IDH] (26/123; no patient showed an IDH mutation) [22]). In the case of recurrence, no further investigations were made. Due to oncological aftercare, adjuvant treatment regimes were recorded in 120 patients. 27 of these 120 patients had combined radiochemotherapy according to the STUPP regime (Fig. 2). EOR was recorded for all patients (complete/incomplete resection of the contrast-enhancing tumor part).

### Statistical analysis

Statistical analysis was performed using IBM SPSS Statistics, Version 24.0 and 26.0 (SPSS Inc., IBM Corp., Armonk, NY, USA). Non-normally distributed data are shown as median and interquartile range (IR), and normally distributed variables are shown as mean and standard deviation. OS was assessed with the Kaplan Meier method in univariate analysis (log-rank). Multivariate survival analysis was performed using a cox proportional hazard regression model. A *p*-value of <.05 was assumed to be statistically significant.

## Results

### Patients and clinical data

The patient population comprises 123 patients (mean age at surgery 57 years, range 21–80 years, 80 male/43 female) with surgery for recurrent GB (Table 1). The median preoperative KPSS was 80% (range 50–100%), and the median postoperative KPSS was 70% (range 0–100%).

**Table 1:** Baseline patient and tumor characteristics

Age	57 years (±10.6)
Sex, female	43/123
KPSS preoperative	80.0 (IR 70.0–90.0)
KPSS postoperative	70.0 (IR 60.0–90.0)
*Tumor hemisphere*	
right	58/123
left	55/123
*ACCI*	
avialable	123,123
median	4.0 (CI 3–6)
*Surgery*	
intraoperative neuromonitoring	87/123
5-ALA	44/123
neuronavigation	109/123
*Extent of resection*	
complete resection	45/123
incomplete resection	78/123
*Adjuvant treatment*	
Stupp scheme	25/123
radiotherapy only	20/123
chemotherapy only	24/123

normally distributed variables shown as mean +/− standard deviation, non-normally distributed as median (interquartile range)

*KPSS* Karnofsky Performance Status Scale

### ACCI and outcome

Median OS was 9.0 months (95% CI 7.1–10.9) after surgery for first recurrence of GB. Median preoperative ACCI was 4.0 (95% CI 3–6).

### Univariate survival analyses

**Fig. 3:**
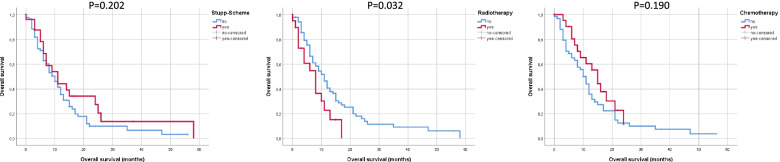
OS according to adjuvant therapy

**Fig. 4:**
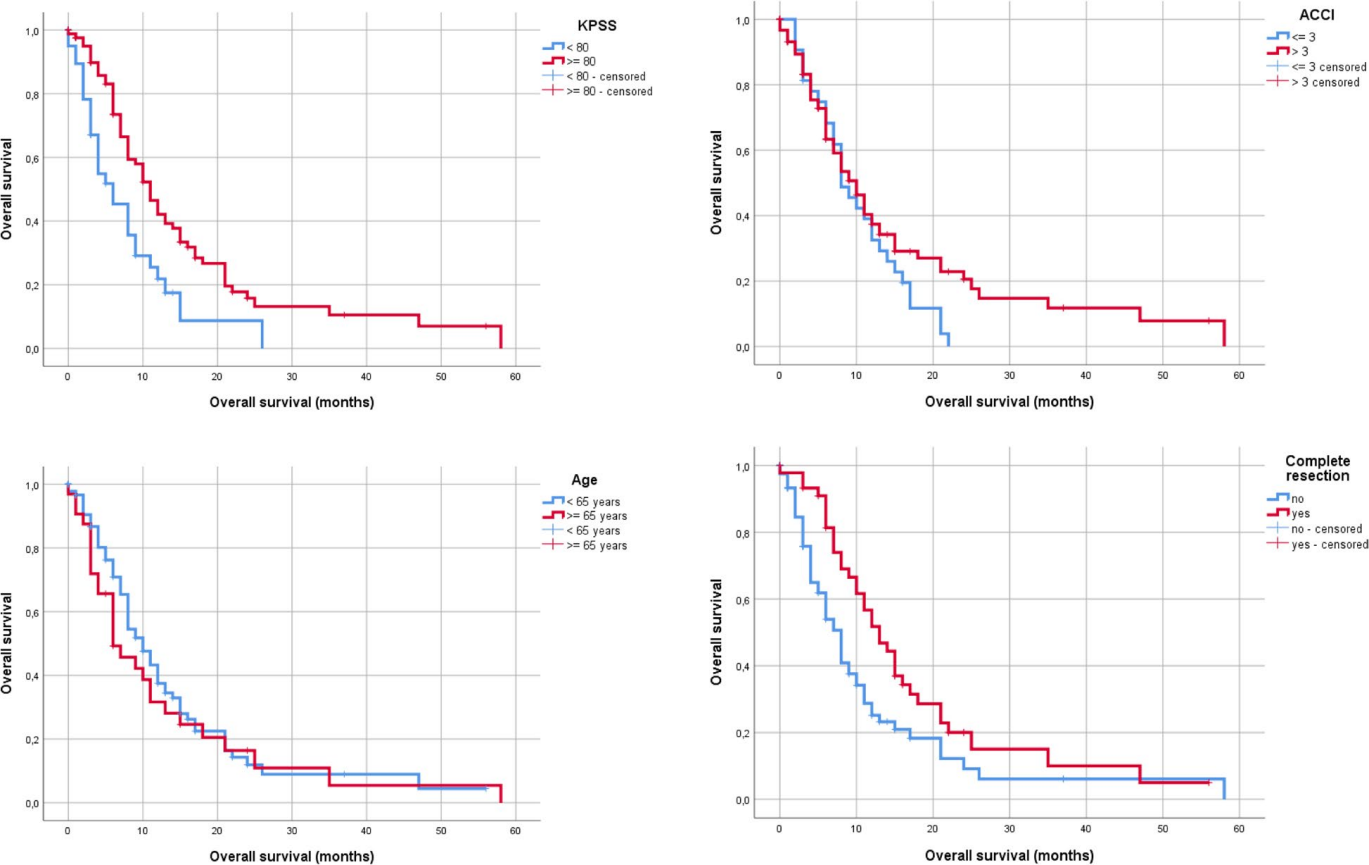
OS according to the age-adjusted Charlson Comorbidity Index (here as an example with cut-off of 3) as well as in relation to the KPSS in GB patients

Preoperative KPSS ≥80% was associated with significantly improved survival after operation for first recurrence of GB (*P* = 0.002). Preoperative ACCI was included in the further analysis as a continuous variable and could not reach statistical significance for survival analysis after the operation for first recurrence of GB. Patients with complete re-resection showed significantly improved survival after surgery (*P* = 0.013). Age, with a cutoff at 65 years, was not significant in relation to OS from operation for first recurrence of GB until death (*P* = 0.445) (Figs. 3 and 4).

### Multivariate survival analyses

Multivariate survival analysis included the following parameters: age (</≥65 years), preoperative KPSS (</≥80), preoperative ACCI, and EOR (complete, incomplete). For survival after the surgery for first recurrence of GB, preoperative KPSS < 80 (HR 2.002 [95% CI: 1.246–3.216], *P* = 0.004) and EOR (incomplete resection vs. complete resection: HR 1.611 [95% CI: 1.036–2.505], *P* = 0.034) were significant prognostic factors. ACCI (HR 0.942 [95% CI: 0.801–1.104], *P* = 0.466) and age < 65 years (HR 0.617 [95% CI 0.339–1.125], *P* = 0.115) did not show significant results. In a further analysis, preoperative KPSS was removed for multivariate analysis, and only the factors age, EOR, and preoperative ACCI were assessed (Table 2).

**Table 2:** Multivariate Cox analysis using ACCI as a categorical variable as well as in dichotomised form

	*Hazard Ratio*	*95% CI*	*P-Value*
Age (> = 65 vs. < 65)	0.62	0.34–1.13	0.12
Postoperative KPSS (< 80 vs. > = 80)*	2.00	1.25–3.22	0.004
Resection (complete/incomplete)	1.61	1.04–2.51	0.034
ACCI	0.94	0.80–1.11	0.467
*A*			
Age (> = 65 vs. < 65)	0.74	0.41–1.32	0.306
ACCI	0.98	0.84–1.15	0.84
Resection (complete/incomplete) *	1.74	1.12–2.67	0.013
*B*			
Age (> = 65 vs. < 65)	0.80	0.50–1.29	0.363
Resection (complete/incomplete) *	1.83	1.17–2.85	0.008
ACCI 6.5	0.64	0.31–1.31	0.219
*C*			
Age (> = 65 vs. < 65)	0.75	0.46–1.21	0.236
Resection (complete/incomplete) *	1.68	1.07–2.64	0.023
ACCI 6.5	0.71	0.34–1.48	0.359
Postoperative KPS (< 80 vs. > = 80)*	1.88	1.18–3.01	0.008

*CI* Confidence Interval; *P</=0.05

A Subanalysis without KPSS; B Subanalysis with the ACCI cut-off of 6.5; C Subanalysis with the ACCI cut-off of 6.5 and KPSS

## Discussion

ACCI might be an additional prognostic factor for patients with recurrent GB; beneath the well-established KPSS, the ACCI, however, did not show statistical significance. The present study assessed 133 patients with surgery or biopsy for recurrent GB. Although there is a standardized treatment for newly diagnosed GB according to a complete surgical resection if possible, followed by concomitant radiochemotherapy as reported in the EORTC Trial [24], all tumors show recurrence. Important prognostic factors in the case of recurrent GB surgery are age, KPSS, and tumor volume, radiation necrosis at time of re-surgery, and the interval between surgeries [6, 25–32]. Contrary to these established prognostic factors, in this study only preoperative KPSS and EOR showed an improved OS after first re-resection. Age missed statistical significance. This might be due to the small study cohort and the fact that young patients are offered a re-surgery more easily than older patients. In addition to these prognostic factors, Park et al. developed a preoperative scale for counseling patients considering repeated surgery and their prognosis. This NIH Recurrent GBM Scale comprised the following characteristics: motor/speech/middle cerebral artery score > 2, KPSS score ≤ 80%, and tumor volume ≥ 50 cm^3^. Each characteristic was assigned one point. They could show that patients with a score of three points expected a 1-month median survival, those with 1–2 points expected an intermediate survival with 4.5 months, and those with zero points presented the best survival with a median of 10.8 months [29]. The KPSS is used for treatment decisions in patients with recurrent GB in clinical practice. Due to demographic changes and an expanding population of elderly with an increasing incidence rate of GB, the patient’s age in the case of relapse is also higher. Treatment concepts for elderly patients with recurrent GB in clinical practice may be individualized by taking into account performance status, response to previous regimens, increasing MGMT promoter methylation status, and quality of life with regard to expected toxicities [33, 34]. Equally, it is apparent that this patient group is usually associated with a higher rate of comorbidities [35]. Therefore, more prognostic factors in addition to the functional status (KPSS) are needed in the preoperative assessment to facilitate decisions in the treatment of these patients. The Charlson Comorbidity Index (CCI) [14] and its further developed age-adapted version (ACCI) [16] are particularly well suited to help classify comorbidities and estimate the risk of death from comorbid disease for use in prognosis. In the present study, the role of ACCI in comparison to KPSS as a prognostic factor in recurrent GB patients was assessed. Several factors, such as surgical resection in relapsing GB, the extent of surgical resection (EOR), and the amount of residual tumor volume, influence the outcomes for these patients [36]. The extent of re-resection and preoperative good performance (KPSS) may serve as independent predictors of survival [37–39]. Likewise, evidence from contrast-enhanced residual tumor volume analysis demonstrated significant OS differences between large (≥15 cc) and small (< 15 cc) tumor sizes in patients in all therapeutic scenarios [40, 41]. Of the many prognostic factors in the case of recurrent GB, in multivariate analysis, preoperative KPSS (≥80/< 80) and EOR were the only significant prognostic factors, whereas ACCI missed statistical significance. In recent studies with relapsing head and neck squamous cell carcinoma, which also showed a poor prognosis with a mean survival of 48 months in the case of a high T stage [42], an ACCI of less than 6 was associated with significantly improved survival [43, 44]. As shown in these studies, we also removed the preoperative KPSS for multivariate analysis, and only the factors age, EOR, and preoperative ACCI were assessed. ACCI might be a prognostic factor, but in comparison to the KPSS, it remains weak. An explanation for this may be that the KPSS is more robust and not, or only marginally, influenced by patient age.

### Limitations of the study

The main limitation of this study is the retrospective design that might introduce an unavoidable bias. The validation of prognostic factors for the outcomes and for supporting therapy selection in recurrent GB would ideally have to be randomized and prospective. Therefore, prospective studies should be performed to assess the value of the ACCI in preoperative patient assessment.

## Conclusion

For patients with surgery for recurrent glioblastoma, the ACCI does not add further information about patient’s prognosis besides the well-established KPSS and extent of resection.

## Data Availability

The datasets used and/or analysed during the current study are available from the corresponding author on reasonable request.
